# DeepDate: A deep fusion model based on whale optimization and artificial neural network for Arabian date classification

**DOI:** 10.1371/journal.pone.0305292

**Published:** 2024-07-30

**Authors:** Nour Eldeen Mahmoud Khalifa, Jiaji Wang, Mohamed Hamed N. Taha, Yudong Zhang

**Affiliations:** 1 Information Technology Department, Faculty of Computers and Artificial Intelligence, Cairo University, Giza, Egypt; 2 School of Computing and Mathematic Sciences, University of Leicester, East Midlands, Leicester, United Kingdom; National Textile University, PAKISTAN

## Abstract

**Purpose:**

As agricultural technology continues to develop, the scale of planting and production of date fruit is increasing, which brings higher yields. However, the increasing yields also put a lot of pressure on the classification step afterward. Image recognition based on deep learning algorithms can help to identify and classify the date fruit species, even in natural light.

**Method:**

In this paper, a deep fusion model based on whale optimization and an artificial neural network for Arabian date classification is proposed. The dataset used in this study includes five classes of date fruit images (Barhi, Khalas, Meneifi, Naboot Saif, Sullaj). The process of designing each model can be divided into three phases. The first phase is feature extraction. The second phase is feature selection. The third phase is the training and testing phase. Finally, the best-performing model was selected and compared with the currently established models (Alexnet, Squeezenet, Googlenet, Resnet50).

**Results:**

The experimental results show that, after trying different combinations of optimization algorithms and classifiers, the highest test accuracy achieved by DeepDate was 95.9%. It takes less time to achieve a balance between classification accuracy and time consumption. In addition, the performance of DeepDate is better than that of many deep transfer learning models such as Alexnet, Squeezenet, Googlenet, VGG-19, NasNet, and Inception-V3.

**Conclusion:**

The proposed DeepDate improves the accuracy and efficiency of classifying date fruits and achieves better results in classification metrics such as accuracy and F1. DeepDate provides a promising classification solution for date fruit classification with higher accuracy. To further advance the industry, it is recommended that stakeholders invest in technology transfer programs to bring advanced image recognition and AI tools to smaller producers, enhancing sustainability and productivity across the sector. Collaborations between agricultural technologists and growers could also foster more tailored solutions that address specific regional challenges in date fruit production.

## Introduction

Regardless of the region, foods are necessary. Agriculture is a fundamental sector that not only meets the needs of the population for food but also has an important impact on the local economy. A wide range of technologies are used in an effort to increase food production [1–3].

Fruits are essential for maintaining a healthy diet, as they provide the body with vitamins and dietary fiber. Therefore, the way to grow, pick, sort, and sell fruit has become a hot topic. Traditional fruit classification methods mainly rely on a huge amount of manual labor. Furthermore, the lack of available human resources has led to an increase in the cost of employing manpower, which has greatly increased the cost of the company. In addition, classification by manual labor is slow and subjective, resulting in low classification accuracy. Using computer vision technology to automatically classify fruit will reduce the need for labor and material resources. Using automatic fruit sorting is an effective solution to increase the efficiency of post-production fruit processing.

Image recognition is a technique that uses computers to process, analyze and understand images to recognise various patterns of targets and objects. The achievements made by deep learning, especially convolutional neural networks, in image recognition, are impressive. Supported by big data and cloud computing, deep learning-based image recognition technology is developing rapidly and is widely used in many fields, such as disease diagnosis [4–8], face recognition [9–12], educational assistance [13–15], security [16–18], and fruit and vegetable classification [19]. Nenad’s proposed new Convolutional Neural Network (CNN) model [20] classifies images of four families, apiaceous, Brassicaceae, Asteraceae, and Apocynaceae, for a total of 52 fruit species. The maximum accuracy of this model was 99.82%. This paper [21] proposed an automated fruit classification using a hyperparameter-optimized deep transfer learning model. In the model, the Recurrent Neural Networks (RNN) model was used to identify and classify the fruit. The Aquila optimization algorithm is exploited for optimal hyperparameters. This paper [22] proposes a multi-model recognition and classification strategy by combining convolutional neural networks, recurrent neural networks, and long and short-term memory. There is no denying that deep learning-based image recognition technology has great potential in the future.

Date trees are an important economic crop in the Arabian Peninsula [23]. Date fruits account for 57% of total fruit production in the Arabian Peninsula [24]. Dates have many effects. Medicinally, Ajwa dates have high nutritional value and have been reported to have antioxidant, anti-inflammatory, and anti-tumour properties [25]. Economically, the date fruit is an excellent source of naturally reducing sugars [26]. The waste and by-products of the process can also be reused as a renewable biofuel [27].

Before artificially intelligent fruit classification is achieved, it is necessary to segment and recognize the fruit images captured in the natural background. However, the morphological position of fruits and vegetables growing in natural scenes is highly random. For many reasons, such as different shooting angles, different lighting conditions, and different shapes due to natural growth, these images are likely to have obscured sagging fruits that make image recognition difficult. Several scholars have proposed methods to solve this problem, which makes it difficult to recognize images of fruit with natural backgrounds. Zhang [28] proposed an image processing algorithm to estimate citrus yields using images of citrus fruits in a natural background. The number of correctly counted fruits reached 91.69%. The target object of Xu’s research [29] is pepper with a high randomness of growth direction. The improved pepper-picking robot of You Only Look Once (YOLOv5s) can identify peppers under different light conditions more accurately. Due to the characteristics of the pineapple, it was unable to select the complete fruit image as the input data for the study. This study proposes an automatic method for identifying pineapple crown image counting using Artificial Neural Network-Gradient Descent ANN-GDX classification detection with an accuracy of 94.4% [30].

The date tree is a very productive fruit tree. Dates are oval in shape and are gathered in clumps of hundreds. Each tree can grow five to ten clusters, each weighing up to seven or eight kilograms. This results in many dates obscuring each other and making them difficult to identify. Although challenging, a number of studies have provided their solutions. The input image used by Albarrak [31] is an image of a single date. The new model proposed in the study uses MobileNetV2 as the architecture. The last layer of the model is replaced by five different layers: the average pooling layer, the flatten layer, the dense layer, the dropout layer, and the softmax layer. Aiadi [32] use the discriminant correlation analysis algorithm to fuse features learned from convolution neural networks and an unsupervised network called Principal Component Analysis Net (PCANet). Unfortunately, the images used in this study were also images of individual dates. Koklu [33] developed the model combining logistic regression and artificial neural networks and evaluated it using a K-fold cross-validation method. The performance result was increased to 92.8%.

Hamdi [34] used a pre-train model Alexnet and Visual Geometry Group (VGG-16) for a date fruit classification model for five types of dates. The Alexnet model achieved an accuracy of 99.01% and VGG-16 achieved 96.5% in testing accuracy. The authors used the same dataset used in this research. However, the results are questionable, and there may be an overfitting problem [35], as the Alexnet model in our experiment achieved 67.29%. That was one of the motivations for doing this research.

In this research, after running DeepDate against some established models on the same dataset, it was found that DeepDate performed better than the Resnet50 model, which achieved the highest accuracy among the established models. In addition to this, it is also competitive with other state-of-the-art methods. DeepDate further improves the accuracy of fruit classification and reduces the time spent on classification. The main contributions of this research are:

1. We have developed a new model named DeepDate specifically for the classification of date fruits. This model uniquely integrates feature selection techniques driven by optimization algorithms with machine learning models, enhancing classification accuracy and efficiency.
2. We introduced a new feature selection layer into the Resnet50 model architecture, utilizing various optimization algorithms to refine this process. This adaptation specifically targets the unique challenges in agricultural data processing, making our approach highly suited for practical applications in date fruit classification.
3. In a novel approach within the field, the standard classification layers of the Resnet50 model were replaced with custom-designed machine learning algorithms to better address the specific needs of date fruit classification, enhancing both accuracy and model adaptability.
4. Our extensive comparisons not only with the original Resnet50 but also with other related works demonstrate that DeepDate provides superior performance. It achieves higher testing accuracy, improves on various performance metrics, utilizes features more efficiently, and reduces both training and testing times.

In the rest of this paper, section 2 introduces the literature background of the proposed model. Section 3 presents the dataset characteristic. Section 4 presents the proposed model architectures and DeepDate. Section 5 illustrates the experimental results. The conclusion and future works will be presented in Section 6.

## Literature background

The model is obtained after going through three phases. The first stage is feature extraction, the second stage is feature selection, and the third stage is the training and testing stage. In the first phase, the feature extractor was selected as Resnet50, which will be described in subsection 2.1. The second phase is the trial of several optimization algorithms, including **w**hale **o**ptimization (WO) [36], **g**rey **w**olf **o**ptimization (GWO) [37], **a**nt **c**olony **o**ptimization (ACO) [38] and **p**article **s**warm **o**ptimization (PSO) [39]. These optimization algorithms will be introduced in subsection 2.2. In the third stage, both support vector networks (SVM) [40] and ANN [41] are attempted in the hope of obtaining the best performance. SVM and ANN will be presented in subsection 2.3.

### Deep learning (Resnet50)

In the field of deep learning, particularly in image recognition, several neural network architectures have set benchmarks for performance. Notable among these are AlexNet, VGG, Inception, and SqueezeNet, each with its own unique advantages and design philosophies. AlexNet, known for its simplicity and effectiveness, was one of the first deep architectures to significantly advance the performance on visual recognition benchmarks. VGG, characterized by its uniform architecture of convolutional layers, is highly regarded for its robustness and was instrumental in popularizing CNNs in image processing.

Inception networks, particularly the GoogleNet series, introduced the concept of parallel convolutions, enhancing the network’s ability to capture information at various scales. SqueezeNet offers a different approach, aiming to reduce the model size and computational expense, which allows for easier deployment on devices with limited resources. These models have been foundational in the development of more advanced architectures, paving the way for innovations that continue to push the boundaries of what is possible in computer vision.

Following these foundational models, more sophisticated architectures like ResNet, DenseNet, and EfficientNet have emerged, each bringing further innovations that address specific challenges in network design and performance. DenseNet, for instance, connects each layer to every other layer in a feed-forward fashion, which is believed to alleviate the vanishing gradient problem, strengthen feature propagation, and reduce the number of parameters. EfficientNet uses a systematic approach to scaling up CNNs in a more structured manner than previous scaling methods, optimizing accuracy and efficiency.

**Fig 1:**
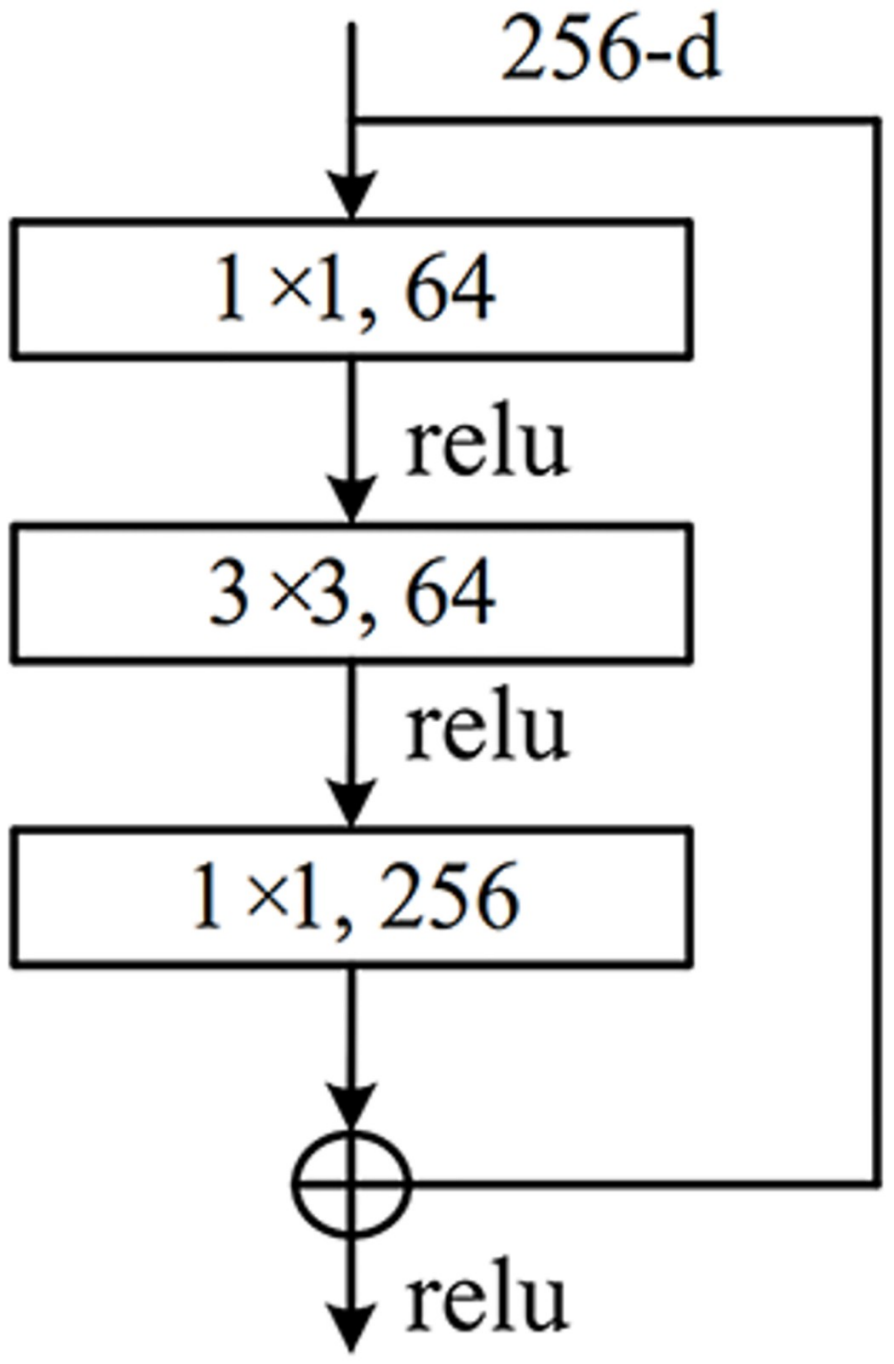
The bottleneck residual block for Resnet50.

In the early stages of CNN development, the number of layers of convolution was increasing in order to obtain deeper features. The early LeNet network [42] only has 5 layers. Then Alexnet [43] was proposed with 8 layers. At a later stage, the Visual Geometry Group Net (VGGNet) network [44] was developed with 19 layers. The more mature and advanced Googlenet [45] has 22 layers. However, it was found that when the number of layers of the proposed neural network model reached a certain number, it would become infeasible to increase the learning capacity of the network by adding more layers. At this point, the network suffers from the problem of vanishing gradients and the accuracy decreases. The Resnet networks are designed to apply the idea of residual learning to the original convolutional neural network to help achieve stochastic gradient descent convergence. This approach keeps a good balance between network depth, performance and speed. In fact, there are two types of residual blocks in Resnet: the basic residual block and the bottleneck residual block. Resnet50 is the most commonly used in Resnet networks and is the one used in this study. The bottleneck residual block is used in Resnet50. The structure of the Resnet50 is shown in the Table 1. The bottleneck residual block is shown as Fig 1.

**Table 1:** The structure of the Resnet50.

Layer name	Output size	Resnet50
Conv1	112×112	7×7, 64, stride = 2
Conv2_x	56×56	3×3 max pool, stride = 2
		$\left[\begin{array}{c} 1\times 1,\;64\\ 3\times 3,\;64\\ 1\times 1,\;256 \end{array}\right]\times 3$
Conv3_x	28×28	$\left[\begin{array}{c} 1\times 1,\;128\\ 3\times 3,\;128\\ 1\times 1,\;512 \end{array}\right]\times 4$
Conv4_x	14×14	$\left[\begin{array}{c} 1\times 1,\;256\\ 3\times 3,\;256\\ 1\times 1,\;1024 \end{array}\right]\times 6$
Conv5_x	7×7	$\left[\begin{array}{c} 1\times 1,\;512\\ 3\times 3,\;512\\ 1\times 1,\;2048 \end{array}\right]\times 3$
	1×1	Average pool, 1000-d fc, softmax

### Optimization algorithms as feature selection

#### Whale optimization

Whale optimization [36] is inspired by the hunting behavior of whales in the ocean. Their favorite prey is krill and small groups of fish. Direct attacks are likely to cause the fish to scatter. When hunting, whales take advantage of their swarming behavior, swimming together and spitting out bubbles to chase together their prey. The most important feature of the whale optimization algorithm is the use of random or optimal individuals to simulate the hunting behavior of whales, and the use of spirals to simulate the bubble net hunting behavior of humpback whales. Based on the hunting behavior of the humpback whale, which swims in a spiral motion toward its prey, the mathematical model of hunting behavior is as follows in Eq (1).

$$X\left(t+1\right)=X^{*}\left(t\right)+D_{p}e^{bl}\;\text{cos}\left(2\pi l\right),$$ (1)

where *D*_*p*_ = |X*(*t*) − *X*(*t*)| denotes the distance between the whale and its prey, X*(*t*) denotes the best position vector so far, *b* is a constant that defines the shape of the spiral and *l* is a random number in (-1,1), *t* is the current number of iterations, *X*(*t*) denotes the current whale’s position vector.

#### Grey wolf optimization

The grey wolf optimization [37] simulates the hierarchy in a natural grey wolf group. The wolves in the first level are the most intelligent and are responsible for leading the entire group in hunting prey, which symbolizes the optimal solution in the optimization algorithm. The wolves in the second level are responsible for assisting the wolves in the first level in their decision-making and are considered to be the best candidates to be the wolves in the first level, symbolizing the sub-optimal solution in the optimization algorithm. The third-level wolf group follows the orders and decisions of the first and second-level wolves, and is responsible for scouting, sentry duty, etc., symbolizing the third-best solution. The wolves in the fourth level do not participate in decision-making and simply follow the wolves in the upper three levels, symbolizing the other candidate solutions. The unique feature of this optimization algorithm is that a small group of grey wolves with absolute say leads the pack towards the prey.

In d-dimensional space, the position of the grey wolf *α* in the first layer is *X*_*α*_ (*X*_*α*,1_, *X*_*α*,2_, …, *X*_*α*,d_), and the position of the grey wolf *i* in the fourth layer is *X*_*i*_ (*X*_*i*,1_, *X*_*i*,2_, …, *X*_*i*,d_). Then, the next position *X*_*αi*_ (*X*_*αi*,1_, *X*_*αi*,2_, …, *X*_*αi*,d_) of the grey wolf *i* under the guidance of grey wolf *α* is calculated as follows in Eqs (2) to (5):

$$X_{\alpha i,\text{k}}=X_{\alpha ,\text{k}}-A_{1}\cdot D_{\alpha ,\text{k}}$$ (2)

$$D_{\alpha ,\text{k}}=\left|C_{1}\cdot X_{\alpha ,\text{k}}-X_{i,\text{k}}\right|$$ (3)

$$C_{1}=2r_{2}$$ (4)

$$A_{1}=2a\cdot r_{1}-a$$ (5)

where *X*_*αi*,k_ denotes the kth component of the spatial coordinate *X*_*αi*_. *D*_*α*,k_ denotes the distance between the grey wolf at *X*_*α*,k_ and the hunted prey. *r*_1_ and *r*_2_ are the random numbers taking values within [0,1]. *A*_1_ is used to simulate the aggressive behavior of the grey wolf towards the hunted prey. *a* is a key parameter for balancing GWO exploration and exploitation capabilities.

#### Ant colony optimization

The ant colony algorithm [38] simulates the behavior of ants in finding the optimal path during their search for food. Ants communicate and cooperate with each other through the pheromones left behind during the searching process and eventually reach cooperation. Each ant emits the most pheromones when it finds food. The further away from the food, the fewer pheromones are emitted. Therefore, if an obstacle is encountered during foraging, ants will choose the direction to walk based on the pheromones. Emulating a colony of ants starting an independent solution search at multiple locations in space at the same time not only increases the reliability of the algorithm but also makes it more capable of global search. The route taken by the ant is determined by the concentration of pheromones. However, the ant does not choose its route based only on the pheromone concentration. The formula for the probability of the ant choosing a route is as follows in Eq (6):

$$P_{ij}^{k}\left(t\right)=\left\{\begin{array}{c} \frac{\tau_{ij}^{\alpha }\left(t\right)*\eta_{ij}^{\beta }\left(t\right)}{\sum_{s\in J_{k}\left(i\right)}\tau_{ij}^{\alpha }\left(t\right)*\eta_{ij}^{\beta }\left(t\right)},\;\;j\in J_{k}\left(i\right)\\ 0,\;\;j\notin J_{k}\left(i\right) \end{array}\right.$$ (6)

$P_{ij}^{k}\left(t\right)$ denotes the probability of ant *k* moving from place *i* to place *j* at time *t*. *α* denotes the relative importance of pheromones. *β* denotes the relative importance of visibility. *τ*_*ij*_(*t*) is the pheromone concentration from *i* to *j* at time t. *η*_*ij*_(*t*) is the reciprocal of the distance between points place *i* and place *j*. *J*_*k*_(*i*) denotes the set of destinations that ant *k* can choose next.

#### Particle Swarm Optimization

**Fig 2:**
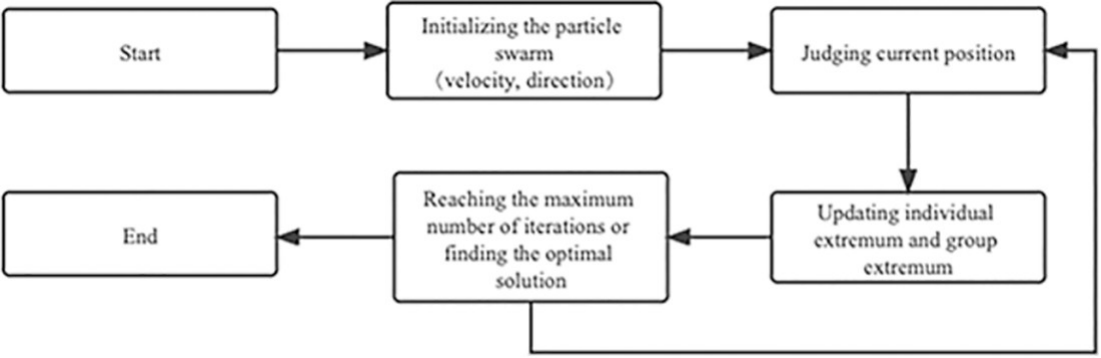
Flow chart of particle swarm algorithm.

Particle Swarm Optimization [39], inspired by the regularity of bird foraging behavior, has the advantage of fast convergence, few parameters, and easy-to-implement algorithms. When no bird in a swarm knows the location of food in the forest, the strategy for finding food is for all birds to search for the bird that is currently closest to the food. In the algorithm, individual birds are represented by particles. The searching process of each particle symbolizes the flight process of the bird. The speed of flight can be dynamically adjusted according to the particle’s historical optimal position and the population’s historical optimal position. The current position of a particle represents a candidate solution. The optimal candidate solution in the particle population is the global optimal solution. The flow chart is as follows in Fig 2.

In the iteration, the velocity and direction of particles are updated as follows in Eq (7)

$$v_{id}^{k+1}=\omega v_{id}^{k}+c_{1}r_{1}\left(p_{id,pbest}^{k}-x_{id}^{k}\right)+c_{2}r_{2}\left(p_{d,gbest}^{k}-x_{id}^{k}\right)$$ (7)

The particle position update formula is as follows in Eq (8).


$$x_{id}^{k+1}=x_{id}^{k}+v_{id}^{k+1}$$ (8)

$v_{id}^{k}$ represents the velocity vector of the *d*th dimension of particle *i* in the *k*th iteration, $x_{id}^{k}$ represents the position vector of the *d*th dimension of particle *i* in the *k*th iteration, *ω* represents inertia weight, *c*_1_ represents the individual learning factor, *c*_2_ represents group learning factor, *r*_1_ and *r*_2_ represent random numbers within an interval, $p_{id,pbest}^{k}$ represents the optimal solution obtained by searching the *i*th particle after the *k*th iteration in the *d* dimension, $p_{d,gbest}^{k}$ represents the optimal solution obtained by the group search after the *k*th iteration in the *d* dimension.

### The classifiers

In routine tasks, people use datasets with defined categories to train classification rules and classifiers, which are then used to classify or predict unknown data. In this paper, we try two classifiers that are now relatively well established.

#### Artificial neural network

Artificial neural network [41] is a very popular deep learning method based on two fields developed in statistics and artificial intelligence. Its most fundamental design idea is to simulate the way the human brain operates to build a non-linear model that treats the input data as features and gets feedback as a result. Artificial neural networks used as classifiers are commonly used in pattern recognition to classify different inputs in order to obtain the correct categorization. Artificial neural networks can change their internal structure based on external information, which is a kind of adaptive system with a learning function. The network structure consists of the input layer, the hidden layer, and the output layer. The backpropagation algorithm continuously calculates the error during the iterations and updates the network and the weights of the corresponding neurons in both the input and output layers.

#### Support Vector Machine

Support Vector Machine [40] is a binary classification model, which belongs to traditional machine learning. For a dataset, the system first generates a random hyperplane and keeps moving to classify the samples until the sample points belonging to different categories in the training sample are located on both sides of the hyperplane. If the dataset is n-dimensional, the hyperplane is (n-1)-dimensional, which splits the linear space into two non-intersecting parts. The main idea is to establish an optimal hyperplane such that the distance between the two classes of samples nearest to the plane on either side of the plane is maximized, thus providing good generalization to the classification problem.

## Dataset characteristics

**Fig 3:**
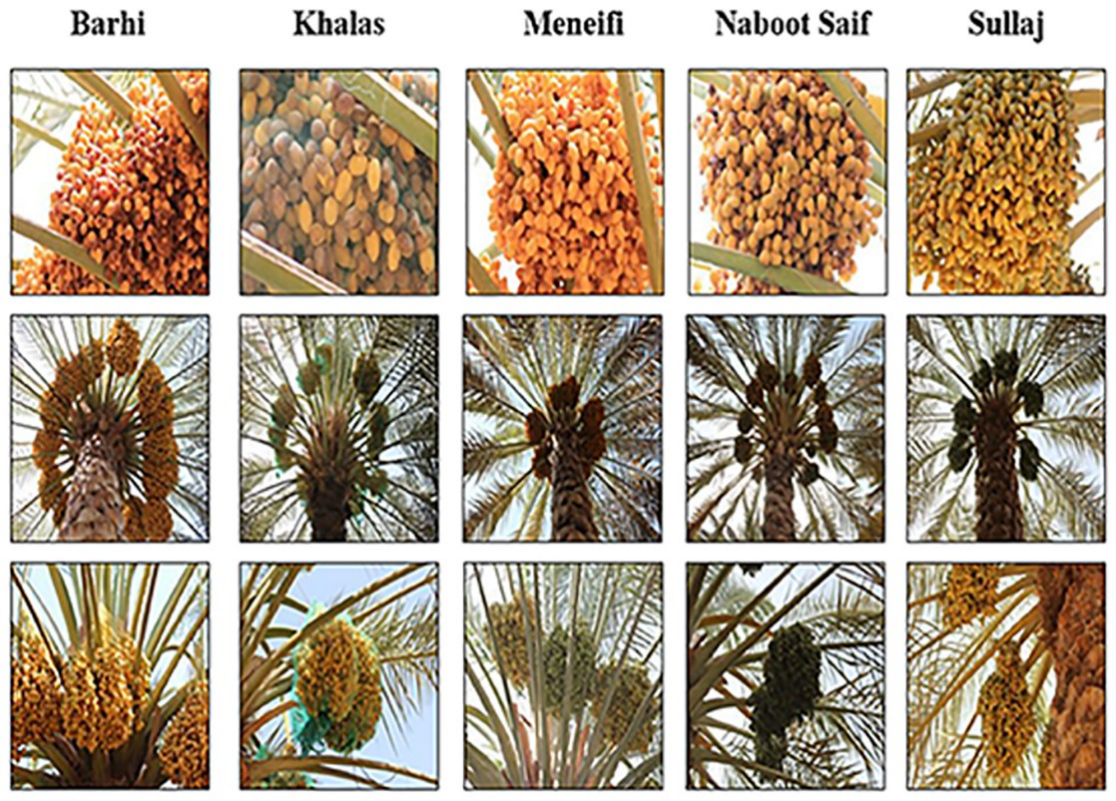
Image examples of date fruit dataset for every class.

The date fruit dataset [46] presented in this research was intended to automate the pre-harvesting and harvesting of dates. It is comprised of two subgroups, each focusing on one of the two primary applications: autonomous harvesting and visual yield estimation. A full description of the environmental conditions, pose conditions, camera characteristics, plant maturity, and growing stage is presented in [46]. This research focuses on the first primary application concerning autonomous harvesting based on date fruit classification. The collection consists of 8072 photos of more than 350 date bunches collected from 29 dates. The date clusters represent five distinct date classes: Naboot Saif, Khalas, Barhi, Meneifi, and Sullaj [46], as illustrated in Fig 3. Six imaging sessions were conducted utilizing a color camera to capture the images. Table 2 presents the number of images for every class in the date fruit dataset.

**Table 2:** Number of images for every class in the date fruit dataset.

Class	Barhi	Khalas	Meneifi	Naboot Saif	Sullaj	Total
**# Images**	1811	1385	1295	1424	2157	8072

## The DeepDate model architecture

To introduce DeepDate architecture, many proposed models’ architectures are designed, validated, and tested. The common design principle is that every model consists of three phases: the first phase is the **f**eature **e**xtraction (FE phase), the second phase is **f**eature **s**election (FS phase), and the third phase is the **t**raining and **t**esting phase (TT phase).

In the FE phase, the features of the different classes’ images are extracted using the **d**eep **t**ransfer **l**earning (DTL) model Resnet50. The selection of Resnet50 as a feature extractor achieves the highest results for accuracy and performance metrics as a DTL model for the classification of the different classes in the dataset. The Resnet50 was compared to other related models such as Alexnet, Squeezenet, and Googlenet. In the experimental section, the results will be illustrated and discussed in detail.

In the FS phase, various optimization algorithms are tested to select the best features of images that allow the architecture to achieve the highest experimental results. The tested optimization algorithms are **w**hale **o**ptimization (WO), **g**rey **w**olf **o**ptimization (GWO), **a**nt **c**olony **o**ptimization (ACO), and **p**article **s**warm **o**ptimization (PSO). WO is selected in the main architecture on the DeepDate as the main FS. The justification for this selection will be presented in the results section.

In the TT phase, the dataset is divided into 80% for the training and 20% for the testing. The division of data on that percentage is commonly used by researchers in the field of deep learning. Also, in this phase, different classifiers, such as SVM and ANN, are tested. The ANN is selected as the main classifier in the proposed model architecture DeepDate as it achieves the highest results. The justification for selecting the ANN classifier will be illustrated in the results section. Algorithm 1 illustrates the selection (nomination) process for the DeepDate model.

**Algorithm 1**. The selection process algorithm of DeepDate model

1: **Input images**: Date fruit dataset images with their **labels** {Naboot Saif, Khalas, Barhi, Meneifi, Sullaj}

2: **Output model**: The model that classifies date fruit based on best performance metrics (DeepDate)

3: best_features = None

4: best_classifier = None

5: best_TA = 0, best_P = 0, best_R = 0, best_F1 = 0

6: **model = load_resnet50_model()**

7: **features = extract_features(model, images)**

8: **optimization_algorithms (*O*) = [WO, GWO, ACO, PSO]**

9:  **foreach** ∀*o* ∈ *O* **do**

10:   **selected_features = select_features(**∀*o***, features)**

11:   **X_train, X_test, y_train, y_test = train_test_split(selected_features, labels, test_size = 0.2)**

12:   **Classifiers (*C*) = [SVM, ANN]**

13:    **foreach** ∀*c* ∈ *C* **do**

14:     **clf = train(**∀*c***, X_train, y_train)**

15:     **TA, P, R, F1 = evaluate(clf, X_test, y_test)**

16:     **if TA > best_TA and P > best_P and R > best_R and F1 > best_F1**:

17:      best_classifier = classifier

18:      best_features = selected_features

19:      best_TA = TA, best_P = P, best_R = R, best_F1 = F1

20:     **endif**

21:    **endfor**

22:   **endfor**

23:  **DeepDate = train(best_classifier, best_features, labels)**

24:  **end**

The dynamic properties of the previous algorithm depend on different factors. The feature selection process dynamic property is the variation in the selected features based on the chosen optimization algorithm, leading to different subsets of features being evaluated for classification. In the classifier selection, the dynamic property is the selection of different classifiers, which can significantly influence the final performance of the DeepDate model. The convergence of optimization algorithms, the dynamic property is the rate of convergence for each optimization algorithm, which affects the overall efficiency of the feature selection phase. In terms of time complexity, the time taken to execute the algorithm can vary depending on the complexity of the optimization algorithms, the number of classifiers, the size of the dataset, and the dimensionality of the feature space. The dynamic property is the variation in the computational time required to complete the feature selection process and train the DeepDate model.

**Fig 4:**
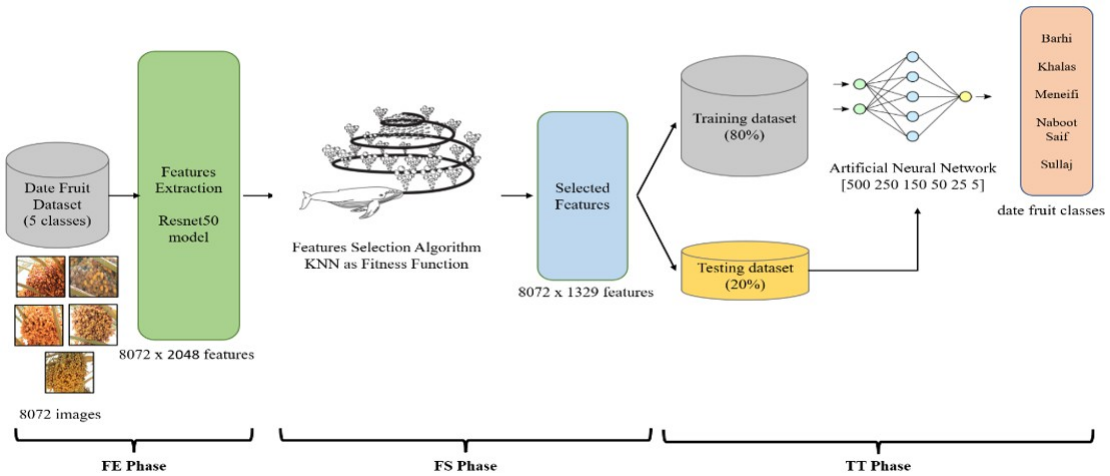
DeepDate model’s architecture design.

Based on the nomination process above. The DeepDate architecture design includes Resnet50 in the FE phase, WO in the FS phase, and ANN in the TT phase. The WO selects 1329 features from the 2048 features that Resnet50 produced. The WO uses the K-Nearest Neighbors Algorithm (KNN) as a fitness function to select the best features that achieve the correct classification of the dataset classes. The ANN architecture includes six layers. The first layers include 500 neurons, which are connected to the selected features (1329) followed by 250 neurons, 125, and 50 neurons. The last layers include five neurons, which reflect the number of classes in the dataset presented in Fig 4.

## Experimental results

The suggested implementation strategy starts with applying different deep transfer models. After that, the deep transfer model that achieves the highest testing accuracy will be selected as a feature extractor in the FE phase. In the FS phase, different optimization algorithms will be tested to select the optimal optimization algorithm that achieves the highest testing accuracy. In the TT phase, different classifiers are tested, and the optimal classifier is selected.

All the experiments were performed on a computer server equipped with 32 GB of RAM, an Nvidia Tesla T4 (16 GB RAM), and a Core i5 processor (3.75 GHz). The development of experiments was GPU-specific on the MATLAB 2021a software package.

### Deep transfer learning models

Four deep transfer-learning models are selected to be tested at the beginning of the experiments. The four DTL models are Alexnet, Squeezenet, Googlenet, and Resnet50. Those models are selected as their architectures include a different number of layers. Moreover, they are commonly used in the area of image classification.

The following hyperparameters are selected during this phase of experiments, and they are:

- Initial learning with stochastic gradient descent with momentum (sgdm) [47].
- The learning rate is 0.001.
- The number of epochs is to be 50, and the early stopping [48] is to be 5 epochs if the accuracy doesn’t improve.
- The mini-batch size [49] is set to 32.
- Classical data augmentation [50] is applied to the dataset images.
- The dataset was divided into two parts [51] (80% of the data for the training and validation process, and 20% for the testing process).

The above hyperparameters were tuned manually by adjusting the hyperparameters and evaluating the model’s performance based on the testing set. The initial hyperparameters were obtained from similar models presented in [52].

Training time and the number of features will be recorded during the experiments. **T**esting **a**ccuracy(TA), **p**recision (P), **r**ecall(R), and **F1** score (F1) [53] are selected as performance metrics and presented from Eqs (9) to (12), along with the consumed time during the training process.

Each metric offers a unique perspective on the strengths and weaknesses of the model. By assessing the percentage of cases that are correctly identified, TA [53] provides an overall assessment of the model’s performance. P denotes the likelihood that a positive occurrence predicted by the model will really occur, lowering the likelihood of false positives. R shows that the model can successfully identify positive occurrences and prevent numerous positives from being missing. A balanced statistic that takes into account both P and R is the F1 score. It helps create a balance between lowering false positives (P) and false negatives (R) because it is the harmonic mean of the two. When the dataset is unbalanced, the F1 score offers a better reflection of the model’s performance [53].

$$\text{TA}=\frac{T_{Po}+T_{Ne}}{\begin{array}{c} \left(T_{Po}+F_{Po}\right)+\left(\;T_{Ne}+F_{Ne}\right)\\ \; \end{array}}$$ (9)

$$\text{P}=\frac{T_{Po}}{\left(T_{Po}+F_{Po}\right)}$$ (10)

$$\text{R}=\;\frac{T_{Po}}{\left(T_{Po}+F_{Ne}\right)}$$ (11)

$$\text{F}1=2*\frac{P*R}{\left(P+R\right)}$$ (12)

Where *T*_*Po*_ is the total number of true positive samples, *T*_*Ne*_ is the total number of true negative samples, *F*_*Po*_ is the total number of false positive samples, and *F*_*Ne*_ is the total number of false negative samples from a confusion matrix.

Table 3 presents the TA, P, R, and F1 for every model along with training and testing time in seconds and the number of extracted features for each model. It also shows that the Resnet50 model achieved the highest accuracy possible in TA, P, R, and F1 with 0.8792, 0.8823, 0.8802, and 0.8812 consequently. In the consumed time for the training and the testing, the Resnet50 model achieves the most consumed time by 2803.85 seconds in training and 7.28 seconds in testing. The time taken for Resnet50 as it is a deeper network than the other models as it contains 177 layers. The Alexnet contains 25 layers, Squeezenet contains 68 layers, and Googlenet contains 144 layers.

**Table 3:** Testing accuracy and performance metrics for different DTL models.

Model/Metric	TA	P	R	F1	Training time (s)	Testing time (s)	# Features
Alexnet	0.6729	0.7006	0.699	0.6998	**747.54**	4.09	9216
Squeezenet	0.7608	0.7779	0.7852	0.7815	1004.20	**3.38**	**1000**
Googlenet	0.8309	0.8438	0.8414	0.8426	1288.34	3.81	1024
Resnet50	**0.8792**	**0.8823**	**0.8802**	**0.8812**	2803.85	7.28	2048

The least consumed time for the training was achieved by Alexnet by 747.54 seconds in the training. The Alexnet contains 25 layers, 61 million parameters and 9216 features. The reason why Alexnet achieved the least time is that it only contains 25 layers and is due to the condition of the early stopping. In the testing time, Squeezenet had the least consumed time, 3.8, as it contains 68 layers, 1.24 million parameters, and 1000 features.

Another metric to be calculated from confusion matrices for the previous DTL models is the accuracy for every class. Table 4 presents the testing accuracy for every class for the different DTL models. The Resnet50 model achieves the highest testing accuracy possible for three classes from five classes. It achieves 0.8890, 0.9470, and 0.7430 for the Khalas, Meneifi, and Naboot Saif class.

**Table 4:** Testing accuracy and performance metrics for different DTL models for every class.

Model/Class	Barhi	Khalas	Meneifi	Naboot Saif	Sullaj
Alexnet	**0.9740**	0.6780	0.6900	0.5120	0.8210
Squeezenet	0.8890	0.8200	0.7790	0.5520	0.8850
Googlenet	0.8320	0.7970	0.9380	0.6490	**0.9910**
Resnet50	0.8410	**0.8890**	**0.9470**	**0.7430**	0.9820

According to previous results, the research decision is selecting Resnet50 as the main feature selection model in the DeepDate model. It achieves the highest accuracy in testing along with performance metrics, and there is still room to improve its accuracy. Moreover, it has a moderate number of features by 2048 that can be reduced in the FS phase and optimize the time consumed in training and testing.

### Feature selection by optimization algorithms

In the FS phase, different optimization algorithms are selected. Those algorithms are **w**hale **o**ptimization (WO), **g**rey **w**olf **o**ptimization (GWO), **a**nt **c**olony **o**ptimization (ACO), and **p**article **s**warm **o**ptimization (PSO) with different classifiers, ANN and SVM. They will compete with each other according to testing accuracy, performance metrics, consumed time in the training, consumed time in the testing, and the number of extracted features. The following parameters are set for every optimization algorithm:

- The fitness function is KNN with K = 5.
- Number of solutions is 10.
- The number of iterations is 50.

Table 5 presents a comparison among different optimizations and classifiers according to TA, P, R, and F1 score for every model, along with training and testing time in seconds and the number of extracted features for each model.

**Table 5:** A comparison among different optimizations and classifiers.

O /Classifier	TA	P	R	F1	FS & Training time(s)	Testing time (s)	# Features
WO—ANN	**0.9591**	**0.9572**	**0.9564**	**0.9568**	462.178	0.032	1329
WO—SVM	0.9536	0.9504	0.9505	0.9505	379.87	0.067	
							
PSO—ANN	0.9511	0.9482	0.9494	0.9488	358.89	**0.014**	979
PSO—SVM	0.9511	0.9488	0.9482	0.9485	366.71	0.057	
							
ANT—ANN	0.9517	0.9497	0.9489	0.9493	439.78	0.018	1240
ANT—SVM	0.9529	0.9496	0.9477	0.9487	471.95	0.056	
							
GWO—ANN	0.9449	0.9433	0.9424	0.9428	**281.27**	0.040	**742**
GWO—SVM	0.9406	0.9375	0.9368	0.9372	284.65	0.064	

From Table 5, it can be concluded that WO with the ANN classifier achieves the highest testing accuracy with performance metrics. Consequently, it achieves 0.9591, 0.9572, 0.9564, and 0.9568 in TA, P, R, and F1. For the FS phase, training GWO with the ANN classifier is required to achieve the least consumed time, with 281.27 seconds. In the consumed testing time, the PSO with ANN classifier achieves 0.014 seconds, and WO with ANN achieves 0.032 seconds. For the number of selected features, GWO achieves the least number of features with 742 selected features, while WO achieves the highest number of selected features with 1329.

So, the research decision was to select WO with ANN classifier to be included in the DeepDate model as it achieves the highest testing accuracy with performance metrics.

### DeepDate model VS Resnet50 model

**Fig 5:**
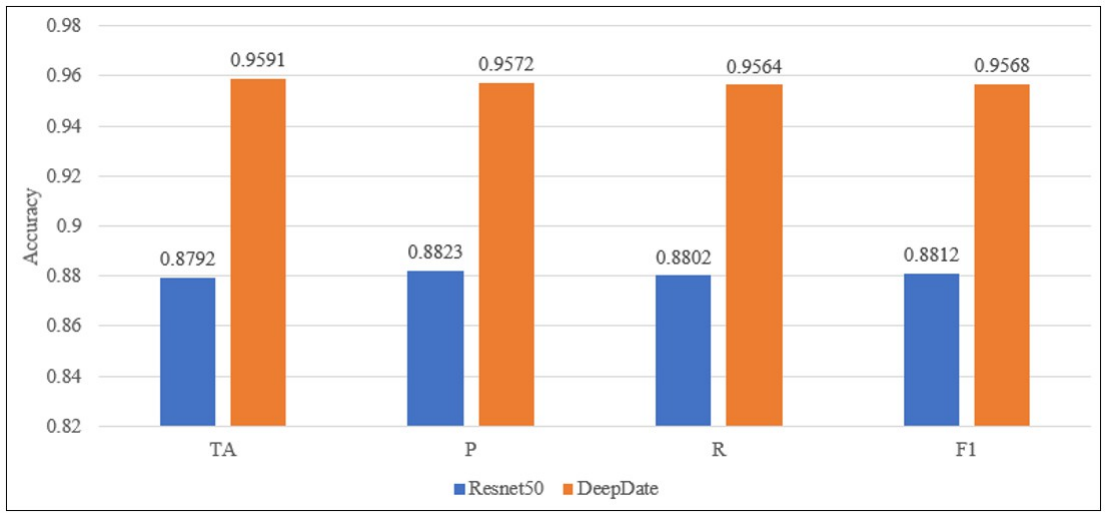
Comparison of Resnet50 and DeepDate according to TA, P, R, and F1 score.

As stated in section 5.1, the Resnet50 model achieved the highest accuracy possible with performance metrics. It overcame the other DTL models (Alexnet, Squeezenet, and Googlenet) in terms of TA, P, R, and F1. This section is dedicated to the comparison between the DeepDate model, and the Resnet50 model. Fig 5 presents a bar graph to compare between DeepDate and Resnet50 according to TA, P, R, and F1.

It is clearly shown that the DeepDate model achieves the highest TA, P, R, and F1 if it is compared to the Resnet50 model. The DeepDate showed an improvement of 7.99% in TA, 7.49% in P, 7.62% in R, and 7.56% in F1 compared to the Resnet50 model.

**Fig 6:**
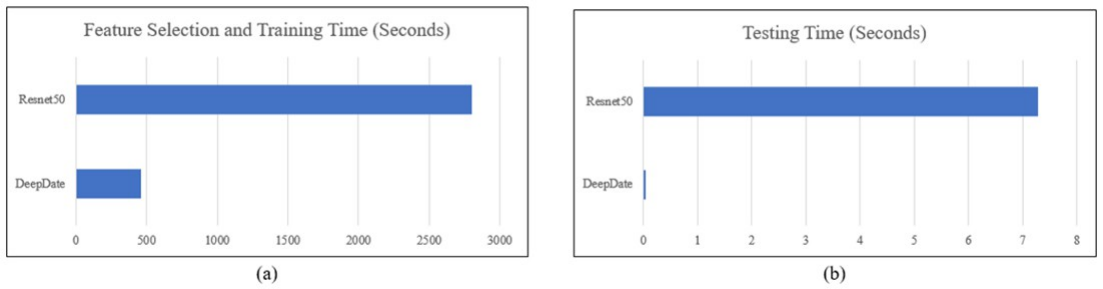
The consumed time for Resnet50 and DeepDate model for (a) FS and training and (b) testing.

Another metric to be in the comparison is the consumed time in the training and the testing. Fig 6 presents the time consumed in the training and testing by DeepDate and Resnet50 Model.

Fig 6 shows that the DeepDate improved the consumed time of FS and training of the Resnet50 model by 2341.68 seconds (83.51% improvement). In the consumed time for testing, the DeepDate achieves 0.032 seconds, while the Resnet50 model achieves 7.28 seconds. For the number of features, DeepDate runs on 1329 features while Resnet50 runs on 2048 features.

The number of features is a key factor in the DeepDate model, which is the reason that the DeepDate consumes less time for training and testing. The ANN classifier is also a key factor in the DeepDate as it is less complicated than the fully connected network in the Resnet50, as stated in the architecture design of the DeepDate.

### DeepDate model vs related work

The DeepDate model proved these competencies against the Resnet50 model and the other DTL models that are investigated in the research, such as Alexnet, Squeezenet, and Googlenet. The DeepDate model achieves the highest accuracy possible in TA, P, R, and F1 and consumes less than the training and testing if compared to the Resnet50 and the other DTL models. The question that may arise is, how about the performance of the DeepDate if it is compared to other related works that use the same dataset?

In [54], the authors investigated other Deep Transfer Learning such as VGG-19, Neural Architecture Search Network (NasNet), Inception-V3, and Resnet. Table 6 presents a comparison with the related work according to TA, P, R, and F1. The DeepDate Model overcame the related work, and it achieved 0.9591, 0.9572, 0.9564, and 0.9568 in TA, P, R, and F1. Meanwhile, the related work achieves less accuracy, regardless of the DTL they used.

**Table 6:** Comparison of DeepDate and the other related work.

Model/Metric	TA	P	R	F1
[54], VGG-19	0.8265	0.8925	0.8650	0.9100
[54], NasNet	0.7750	0.9280	0.7780	0.8375
[54], Inception-V3	0.8340	0.9290	0.8330	0.8680
**DeepDate (Ours)**	**0.9591**	**0.9572**	**0.9564**	**0.9568**

The author of [54] claimed that they used the Resnet model and achieved 0.99% in TA, P, R, and F1. But they never mentioned which type of Resnet they used. Is it Resnet18, Resnet50, or Resnet101? This result is questionable and doubtful, and it may be an overfitting problem [35], so we decided not to include it in the comparison table. The only explanation for their results is they might use Resnet101, which is a deeper network (more layers) than Resnet50, and hence it will take too much time in training and testing and consume a lot of computer resources to achieve 3% percent more than the DeepDate in the testing accuracy.

**Fig 7:**
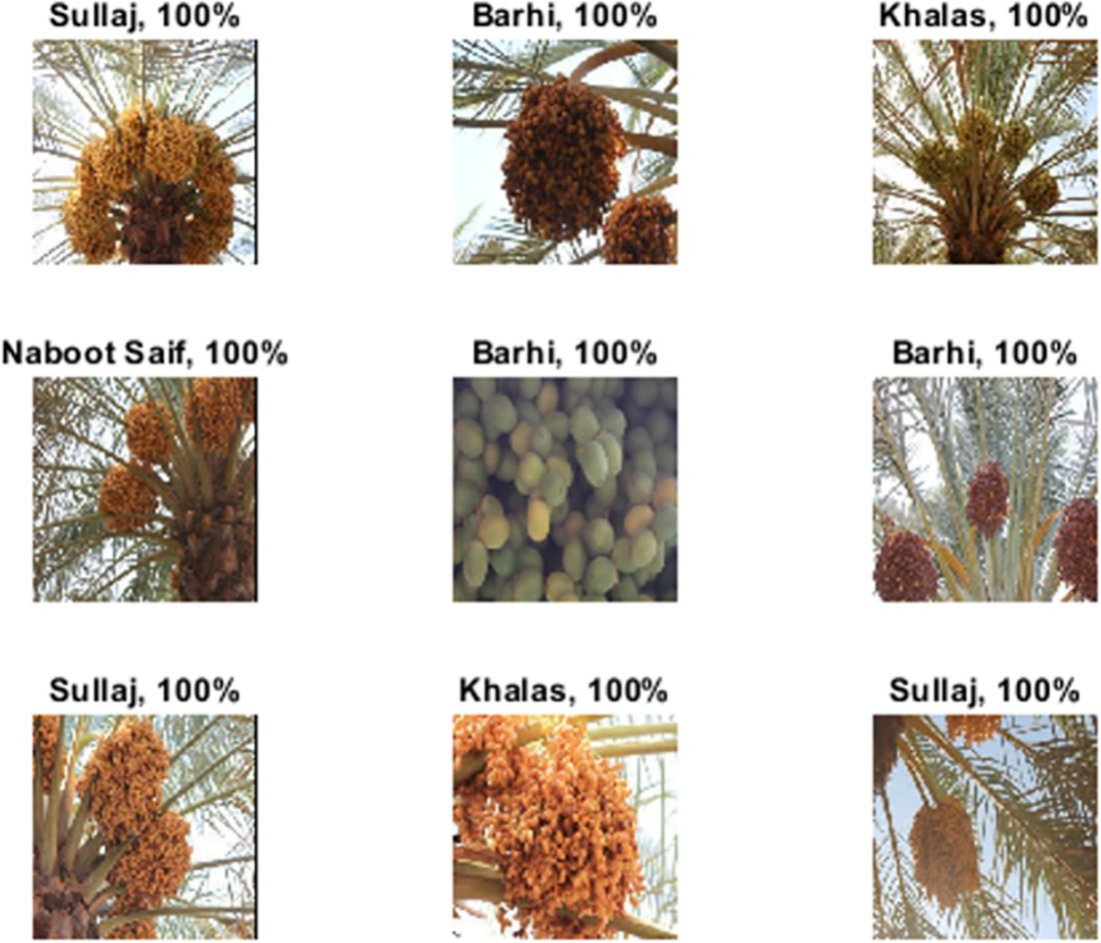
Random sample of dataset image with the testing accuracy for every image with different classes.

In the end, the DeepDate proved its performance against many DTL models such as Alexnet, Squeezenet, Googlenet, VGG-19, NasNet, and Inception-V3 and improved the time consumed in the training and the testing and run-on fewer features and hence it consumes less memory and achieves very competitive results in terms of TA, P, R, and F1 score. Fig 7 presents a random sample of the out of the DeepDate model with the testing accuracy above every image.

One of the possible limitations of the DeepDate is the limitation of the deep learning models itself, such as interpretability. Deep learning models are (black boxes) because they lack transparency and interpretability. Understanding how and why the model makes certain predictions can be a challenging task. In the field of increasing yield for Arabian date fruit, there are some challenges, such as the existence of new Arabian date fruit varieties that were not present in the original dataset used to train the DeepDate. If the DeepDate model encounters out-of-distribution data (unseen or novel varieties), it may struggle to classify them accurately.

## Challenges and future works

The DeepDate model encounters various challenges and limitations that necessitate careful consideration and future research. One primary challenge is the morphological changes that dates undergo due to diseases or regional variations, leading to different appearances for the same type of date under varying conditions. To address this issue, we propose using larger and more diverse datasets, encompassing a wider range of image types, to enhance the model’s adaptability to different appearances of Arabian dates.

Another limitation lies in the scale and diversity of the Arabian date dataset. Creating a comprehensive and diverse dataset for Arabian dates is challenging due to the numerous date varieties, each with various variants. To overcome this limitation, we plan to actively collect and integrate data from different sources and regions, collaborating with other research institutions or communities to promote dataset diversity.

Data preprocessing is a crucial aspect of improving the model’s accuracy. Proper data preprocessing can remove image noise, standardize image sizes, and colors, enhancing the model’s robustness and performance. Furthermore, employing visualization techniques will increase the model’s usability. By visualizing the model’s decision-making process, we can better understand its behavior and identify potential misclassification cases, guiding further optimization and improvement efforts.

Additionally, we aim to extend the application of the DeepDate model to other fruit classification tasks, leveraging its advantages and experiences for a broader range of applications, thus enhancing its versatility and practicality.

One specific limitation worth mentioning is the model’s struggle to classify out-of-distribution data, such as unseen or novel varieties of Arabian date fruit. Exploring the impact of different data augmentation techniques on model performance and investigating alternative optimization algorithms and feature selection techniques could be potential areas for future research to further improve accuracy and efficiency.

Integrating explainable AI (XAI) features into the DeepDate model directly tackles the opacity often found in deep learning frameworks. By making the decision-making processes clearer, XAI empowers stakeholders like farmers and agricultural technicians, fostering trust and simplifying model adjustments and troubleshooting.

Further, acknowledging the repercussions of climate change on agriculture, the integration of climatic data is important. By predicting how date fruit morphologies may shift under varied environmental pressures, we can enhance the model’s adaptability and resilience. This proactive approach not only buffers the model against morphological variations but also supports the development of robust strategies for crop sustainability.

Moreover, linking the DeepDate model with Geographic Information Systems (GIS) enhances its utility in precision agriculture. This combination facilitates precise geographic tracking of date fruit varieties and their conditions, enabling optimized resource management and strategic planning based on detailed spatial analysis of crop health. These technological integrations transform the DeepDate model into a sophisticated, multifaceted tool that aligns with both contemporary technological trends and pressing environmental challenges, making it an invaluable asset in the evolving landscape of agricultural practices.

Moreover, it would be intriguing to compare the performance of DeepDate on different types of date fruit or even other types of fruits and vegetables, broadening the model’s scope and potential applications. By addressing these challenges and pursuing further research, the DeepDate model can reach new heights in date fruit classification and potentially extend its impact to other domains of food classification and beyond.

## Conclusion

Arabian Date fruit, which is widely cultivated in arid and semi-arid regions around the world, is an important source of nutrition and income for many communities. As the demand for date fruit grows, so too does the need for efficient and accurate methods of classifying different types of date fruit. One promising approach is the use of image recognition algorithms based on deep learning, which is capable of accurately identifying and classifying date fruit species even in natural light.

In this paper, the DeepDate model has been introduced and proven to be a promising solution for the classification of date fruit. The model is based on a deep fusion approach that combines whale optimization and artificial neural networks, and it was trained on a dataset containing images of five different types of date fruit. The results of the experiments showed that DeepDate achieved the highest accuracy among the models tested, with an accuracy of 95.9%. The DeepDate model proved its performance against many well-established DTL models such as Alexnet, Squeezenet, Googlenet, VGG-19, NasNet, and Inception-V3. In addition, DeepDate was able to achieve this high level of accuracy in a relatively short amount of time, indicating that it is an efficient model for date fruit classification.

Despite its success, DeepDate did encounter some challenges. In light of these challenges, further research will explore the scalability of DeepDate to larger, more diverse datasets to enhance its robustness and applicability. Further investigation into visualization techniques will also be pursued to better understand the model’s decision-making process. Additionally, we aim to extend the DeepDate model to classify other fruit types, which could provide a comprehensive solution for the agricultural sector and support sustainable farming practices by improving yield quality assessment and crop monitoring.

As the demand for date fruit continues to grow, the potential of the DeepDate model to be integrated into automated harvesting systems could significantly contribute to enhancing date fruit classification methods and benefiting communities reliant on this valuable agricultural resource.
